# Shear Bond Strength and Film Thickness of a Naturally Antimicrobial Modified Dental Luting Cement

**DOI:** 10.3390/molecules26051276

**Published:** 2021-02-26

**Authors:** Lamia Singer, Christoph P. Bourauel

**Affiliations:** Oral Technology, University Hospital Bonn, 53111 Bonn, Germany; bourauel@uni-bonn.de

**Keywords:** medicinal plants, dental luting cement, shear bond strength, film thickness

## Abstract

Although several natural plants and mixtures have been known and used over the centuries for their antibacterial activity, few have been thoroughly explored in the field of dentistry. Thus, the aim of this study was to enhance the antimicrobial activity of a conventional glass ionomer cement (GIC) with natural plant extracts. The effect of this alteration on the bond strength and film thickness of glass ionomer cement was evaluated and related to an 0.5% chlorohexidine modified GIC. Olive leaves *(Olea europaea)*, Fig tree (*Ficus carica)*, and the leaves and roots of Miswak (*Salvadora persica)* were used to prepare an alcoholic extract mixture. The prepared extract mixture after the evaporation of the solvent was used to modify a freeze-dried glass ionomer cement at three different extracts: water mass ratios 1:2, 1:1, and 2:1. An 0.5% chlorhexidine diacetate powder was added to a conventional GIC for the preparation of a positive control group (CHX-GIC) for comparison. The bond strength to dentine was assessed using a material-testing machine at a cross head speed of 0.5 mm/min. Failure mode was analyzed using a stereomicroscope at 12× magnification. The cement film thickness was evaluated in accordance with ISO standard 9917-1. The minimum number of samples in each group was *n* = 10. Statistical analysis was performed using a Kruskal–Wallis test followed by Dunn’s post hoc test for pairwise comparison. There was a statistically insignificant difference between the median shear bond strength (*p* = 0.046) of the control group (M = 3.4 MPa), and each of the CHX-GIC (M = 1.7 MPa), and the three plant modified groups of 1:2, 1:1, 2:1 (M = 5.1, 3.2, and 4.3 MPa, respectively). The CHX-GIC group showed statistically significant lower median values compared to the three plant-modified groups. Mixed and cohesive failure modes were predominant among all the tested groups. All the tested groups (*p* < 0.001) met the ISO standard of having less than 25 µm film thickness, with the 2:1 group (M = 24 µm) being statistically the highest among all the other groups. The plant extracts did not alter either the shear bond strength or the film thickness of the GIC and thus might represent a promising additive to GICs.

## 1. Introduction

During the 19th century, amalgam and gold were commonly used as restorative materials in dental treatments. Nevertheless, their unsatisfactory color created the need for more aesthetically acceptable dental cements and restorative materials [1]. Glass ionomer cements (GIC) were one of the crucial steps in this direction and have become one of the most commonly used restorative materials in dentistry [2,3] GICs are byproducts of an acid–base reaction between weak polyacrylic acids and aluminosilicate glass powder. The set cement contains unreacted glass particles which play a role in in reinforcing the final cement structure [4,5]. Glass ionomer-based cements are the material of choice for cementation, liners, bases, atraumatic preventive treatments, and restoring cervical dental lesions [6,7]. They have the advantage of forming a chemical adhesion with the tooth structure, thus requiring minimal preparation, fluoride release, biocompatibility, antimicrobial activity, recharge ability, and reverse potential to reduce the acidic environment [8,9,10].

The success of dental materials clinically depends on many factors, among which is the good adhesion to the surface of the tooth to resist various dislodging forces [11]. Shear bond strength is known as the resistance to dislodging forces, which causes the sliding of the restorative material against the tooth structure. It adopts much importance to the dentist clinically because it has been proven that the major dislodging forces at the tooth restoration interface have a shearing effect [12,13].

Along with the mechanical properties for the selection of a suitable and durable luting agent, there are other clinically related properties that need to be taken into consideration, such as the film thickness [14]. During cementation, achieving a minimum film thickness is very important for the complete seating and adaptation of the prosthetic restorations. Moreover, a thin film thickness decreases the marginal discrepancies, cement dissolution, plaque accumulation, and periodontal disease [14,15].

The use of herbal products is increasing at an exponential rate in both developing and developed countries owing to the free availability, religious beliefs, as well as unique chemical composition [16]. This novel branch has its roots in ancient medicine and the pre-antibiotic era. Herbal extracts were claimed to have the advantage of showing their beneficial effects without the risk of developing microbial resistance. Nowadays, several herbal products are available in the market in different forms, such as toothpastes, oral gels, and mouth rinses [17,18,19].

*Salvadora persica (S. persica)* is a small tree that belongs to the family Salvadoracea and is commonly known as miswak (toothbrush) tree. Studies of miswak against oral bacteria such as *Streptococcus mutans, salivaris, Staphylococcus aureus*, and *mitis* have proven that the crude extract was significantly effective, with an inhibition zone production of 67 and up to 96% [20,21].

*Ficus carica (F. carcis)* belongs to the family *Moraceae and* is commonly referred to as “Fig”. Several authors have claimed that *F. carica* has antioxidant, antiviral, antibacterial, hypocholesterolemia, hypoglycemic, cancer-suppressive, and hypotriglyceridemic effects [22,23].

*Olea europaea (O. europaea)* leaves and olive fruits have an ancient history of therapeutic and traditional practices. The olive tree, leaves, and extracts are an essential part of the Mediterranean culture due to olive polyphenols. Olive leaf polyphenols have been thoroughly investigated because of their anti-inflammatory and antimicrobial activities and anti-hypertensive, anti-diabetic, anti-carcinogenic, and anti-atherosclerotic potentials [24,25].

While many studies support the notion of the protective effect of fluoride in public water and oral health products, the available data still do not endorse the anti-caries ability of fluoride-releasing restorative materials such as GIC [26,27]. Based on the ability of GIC to participate in ion-exchange reactions with the oral environment, many modifications have been carried out to improve its antimicrobial properties [26,27].

In earlier study, an extract mixture of *S. persica, F. carcia,* and *O. europaea* incorporated in a conventional GIC showed a significant antimicrobial activity against *Streptococcus mutans* and *Micrococcus luteus*. Moreover, the chemical characterization of the extract mixture using GC/MS has shown many chemically active compounds, including phenols, flavonoids, alkaloids, carboxylic acids, terpenes, and more [28].Despite the recommendations for the use of these herbal plant extracts, there are only a few available studies that involve the addition of natural herbal extracts to GIC. Additionally, the antimicrobial effects were the focal point of these studies, while the physical-mechanical properties have been overlooked. Thus, the aim of this study was to evaluate the shear bond strength and film thickness of a GIC modified with a natural plant extract, while a 0.5% CHX-modified GIC (positive control) and an unmodified GIC were used for comparison. The null hypotheses were there will be no significant difference between the extract-modified groups, the CHX-modified group, and the control with regard to shear bond strength, failure mode analysis, and film thickness.

## 2. Results and Discussion

### 2.1. Results

#### 2.1.1. Shear Bond Strength

**Figure 1:**
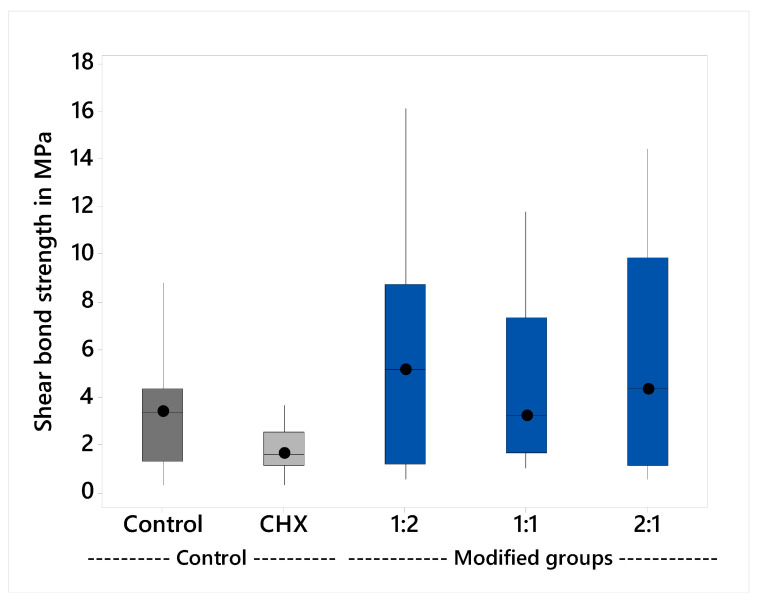
Median shear bond strength and interquartile range.

The variables showed a non-parametric distribution and thus the Kruskal Wallis H test was used to test the effect of the plant extract on the shear bond strength. The results are shown in Table 1 and illustrated graphically in Figure 1. The Kruskal Wallis H test indicated that there was significant difference between the groups, *p* = 0.046. Post hoc comparisons using Dunn’s test showed significant differences between the CHX-GIC shear bond strength (M = 1.7 MPa) and the modified groups 1:2, 1:1 and 2:1. However, there were insignificant differences between the control groups and all other groups.

##### Failure Mode 

**Figure 2:**
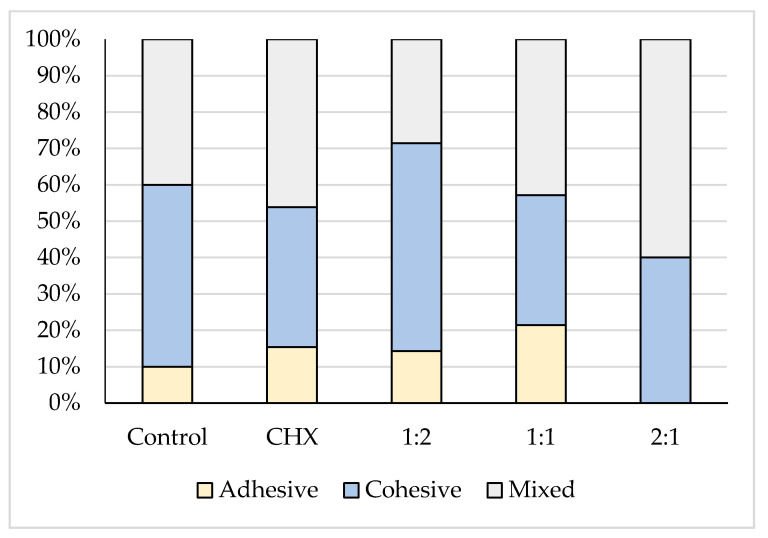
A stacked column chart showing the different failure modes of the five tested groups.

The stereomicroscope examination of the deboned dentin surface after shear bond strength testing revealed that the majority of the fracture modes were cohesive and mixed failure, as presented in Table 2 and illustrated in Figure 2.

#### 2.1.2. Film Thickness

**Figure 3:**
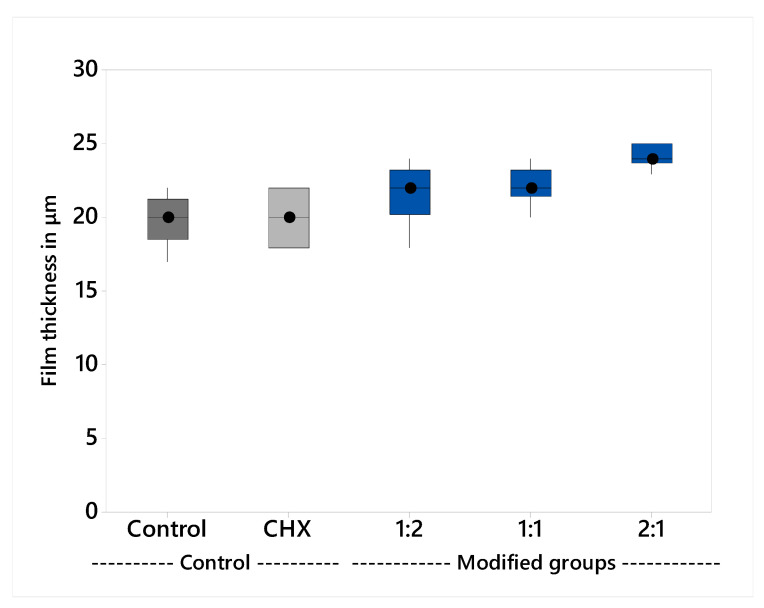
Median film thickness and interquartile range.

The variables showed a non-parametric distribution and thus the Kruskal Wallis H test was used to test the effect of the plant extract on film thickness; the results are shown in Table 3 and illustrated graphically in Figure 3. The Kruskal Wallis H test indicated that there was significant effect of the plant extract on the film thickness, H (4) = 27.3, *p* < 0.001. The group 1:2 (M = 24 μm) had the thickest film and the post hoc comparisons using Dunn’s test showed that it was significantly different from all groups, except group 1:1. All the other groups had insignificant differences compared to each other.

### 2.2. Discussion

Despite the common usage of glass ionomer cement (GIC) in dentistry because of the anticariogenic property, fluoride release, and rechargeability, the reduction in the bacterial counts and the ability of the conventional glass ionomer cements to completely arrest the caries process is still not reliable for many clinical situations. Therefore, many investigations are concerned with improving the antibacterial activity of GIC to overcome this problem [29,30].

GIC modified with *S. persica*, *F. carcia,* and *O. eoropaea* extract mixtures has shown significant antimicrobial activity against *S. mutans* before, which is the main causative organism of dental caries and *M. luteus*, which is a sensitive marker to the release of antimicrobial agents [28]; thus, this study aimed to assess two important clinical properties of GIC, which are shear bond strength and film thickness.

#### 2.2.1. Shear Bond Strength

Clinical success and the retention of a dental cement are directly affected by its adhesion and bonding to the tooth structure. The mechanism of adhesion of glass ionomer cement to the tooth structure was attributed to the interaction of hydroxyapatite found in the tooth structure with the polyacrylic acid forming strong ionic bonds [31,32,33].

The bond strength assessment of GIC may be influenced by several factors: testing device, size of the specimen, composition of the tooth structure, storage time, temperature, and the substrate [34]. Enamel is much more susceptible to adhesion than dentin, where values of enamel vary between 2.6 to 9.6 MPa and values of dentin vary from 1.1 to 4.1 MPa [4,35]. Enamel has a surface that is basically homogeneous, and mainly composed of hydroxyapatite, which has high surface energy, whereas dentin has a heterogeneous surface with low surface energy [36]. Moreover, it was found that GIC recorded lower bond strength values to tricalcium silicate-based cements compared to methacrylate- and silorane-based composites [37].

The shear bond strength test in the current study was carried out after 24 h because it was found that bond strengths increase rapidly, with about 80% of the final bond strength being achieved in the first 15 min [4,38]. The results showed that there were no significant differences among the median values of control (M = 3.7 MPa) and CHX-GIC (M = 1.7 MPa). Likewise, there was insignificant difference between the control and the three extract-modified groups: 1:2 (M = 5.1 MPa), 1:1 (M = 3.2 MPa), and 2:1 (M = 4.3 MPa) groups. This could be due to the amount of CHX (0.5%) added to the CHX-GIC group, and the amount of plant extract in the 1:2, 1:1, and 2:1 groups did not negatively alter or affect the ionic exchange and interaction between the cement and the surface of the tooth. This was in accordance with the results of Becci et al. [39] and Jaidka et al. [40].

On the other hand, the CHX-GIC group showed statistically significant lower median values compared to all the plant-modified groups (1:2, 1:1, 2:1). The reason for this could be due to the presence of Cinnamic and bornyl acetic carboxylic acids in the plant extract mixture [28]. According to Prentice et al. [41, those carboxylic acids might have been existed in a considerable amount that improved release of ions from the surface of the glass ionomer powder through lowering the pH. Moreover, the presence of additional COOH groups from acids might have caused more ionic exchange and interaction with calcium of the tooth within first 24 h. This might explain the slight potential enhancement of the bond strength specifically in group 1:2 (M = 5.1 MPa) compared to CHX, but still it is statistically insignificant compared to the control group (M = 3.7) [41].

##### Failure Mode

Dental restorations and cements should ideally have high adhesive and cohesive bond strengths to counteract the forces of mastication [42]. In the present study, the deboned dentine surface was observed using stereomicroscope at a 12× magnification in which cohesive and mixed patterns predominated. Choi et al. [43] and Becci et al. [38] accounted cohesive failure prevalence for a low tensile strength of the tested GIC material rather than its true adhesive bond strength to dentin. Lucas et al. [44] attributed this to the strong ionic layer that is formed at the interface between the GIC cements and the calcified structures through an ion exchange process.

For the mixed failure, Palma-Dibb et al. [45] and Carvalho et al. [46] explained it on the basis of the insufficient resistance to early wear and the formation of a glass ionomer matrix. Therefore, part of the glass ionomer remained bonded to the tooth structures, while part was dislodged at the GIC–tooth interface. No correlation was found in the present study between the shear bond strength values and failure modes, because this correlation has been discussed controversially in the literature [47,48]. El Wakeel et al. [49] indicated that there is no relationship between the shear bond strength and the mode of failure.

#### 2.2.2. Film Thickness

Glass ionomer cements have been used widely for the cementation of cast metal and porcelain restorations in dentistry [50]. Film thickness is a significant rheological property that should be taken into consideration during the selection of a suitable and durable luting agent. Film thickness is highly influenced by manipulation variables, such as mixing temperature and powder–liquid ratio. The consistency of the luting cement directly affects the film thickness and the correct adaptation of the restoration. A luting material with a high viscosity requires more time for the optimal seating of the restoration as well as the application of higher seating forces to prevent marginal gaps [51,52].

Film thickness was evaluated consistent with ISO 9917-1. The results showed that all the groups meet the standard, with less than 25 µm film thickness [53]. There was a statistically insignificant difference in the mean values between the control group (M = 20 µm), CHX-GIC (M = 20 µm), and the plant modified groups; 1:1 (M = 22 µm), 1:2 (M = 22 µm). The 2:1 (M = 24 µm) group showed statistically significantly higher mean values compared to all the other tested groups. The results were in agreement with those of Sulaiman et al. [54] and Kious et al. [55]. This could be explained on the basis that the plant extract mixtures did not alter the viscosity of GIC, which directly affects the cement film thickness, where cements of high viscosity showed rapid setting before they can flow properly to achieve a minimum film thickness [56].

The null hypotheses of both shear bond strength and film thickness were rejected based on the results. A limitation of the current study is that it was designed as an in vitro study and thus the testing conditions did not exactly simulate the oral environment and the clinical situations. Different factors affect the physical and mechanical properties of GIC, such as moisture contamination, the application of a protective coat, mixing time and temperature, batch of cement, and storage medium [57]. Further studies with respect to other bacterial strains and more mechanical and physical properties will be performed.

## 3. Materials and Methods

### 3.1. Plants Extraction and GIC Modification

#### 3.1.1. Plant Extraction

Three different plants, *Olea europaea* leaves, *Ficus carcia* leaves, and *Salvadora persica* roots, were washed thoroughly with water, dried in air for 6 days at room temperature, and ground using a blender into a fine powder. A standardized amount (80 g) from each plant powder was placed into a Soxhlet extractor (Carl Roth GmbH + Co. KG, Karlsruhe, Germany) separately and an extraction process was carried out using 250 mL of ethyl alcohol (70%) at 75 °C. The resultant product of each process was then filtered using Whatman filter paper no. 1 and mixed together to prepare an extract mixture. A rotary evaporator (Buchi Rotavapor R-300, Buchi Labor Technik GmbH, Essen, Germany) was used to evaporate the solvent at 37 °C, leaving a concentrated crude mixture that was stored at 4 °C in a glass bottle until usage [58].

#### 3.1.2. Modification, Preparation and Specimens Grouping of GIC

Conventional freeze-dried glass ionomer cement (Medicem aqua, Promedica GmbH, Neumuenster, Germany, Lot 1849261) that was supplied in the form of powder/water version was used. The distilled water used for the preparation of GIC was modified with the extract mixture at three different extracts of water mass ratios, giving three plant-modified groups (1:2, 1:1, 2:1). Plastic bottles with the exact nozzle size as those supplied by the manufacturer were used to store the different groups in order not to alter the recommended powder/liquid ratio (1:2) upon cement preparation. Fresh specimens of each of the modified groups were prepared according to the recommended powder/liquid ratio (1:2) for each testing procedure and then compared with two control groups:

- Negative control: prepared by mixing the powder of GIC with the exact amount of distilled water as per the manufacturer’s instructions (1:2), without any modification.
- Positive control: prepared by adding 0.5% CHX diacetate powder (*w*/*w*) (Merck KGaA, Darmstadt, Germany) to GIC powder (CHX-GIC) to be mixed with distilled water (1:2).

Group names:

1. Control: (unmodified GIC).
2. CHX-GIC: (0.5% CHX modified GIC).
3. Extract- mixture modified groups:
  - 1:2 (extract: water).
  - 1:1 (extract: water).
  - 2:1 (extract: water).

### 3.2. Shear Bond Strength

**Figure 4:**
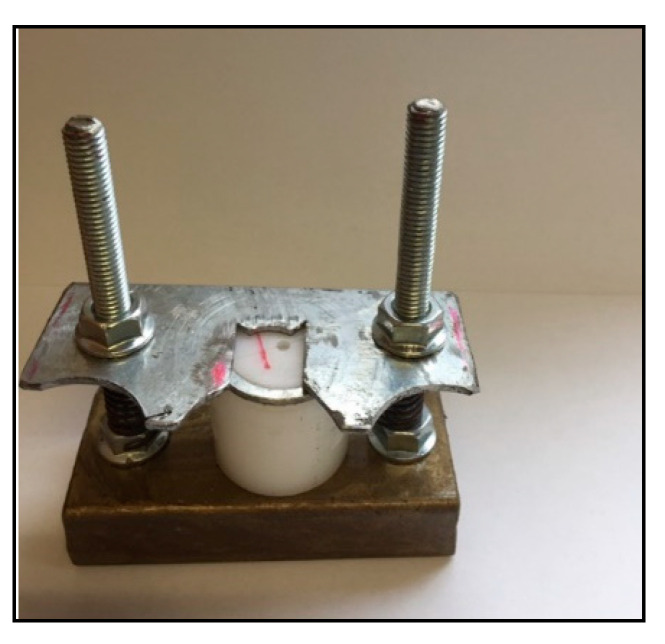
Teflon mold fixed by a metallic device with screws on the tooth surface for bond strength specimen preparation.

**Figure 5:**
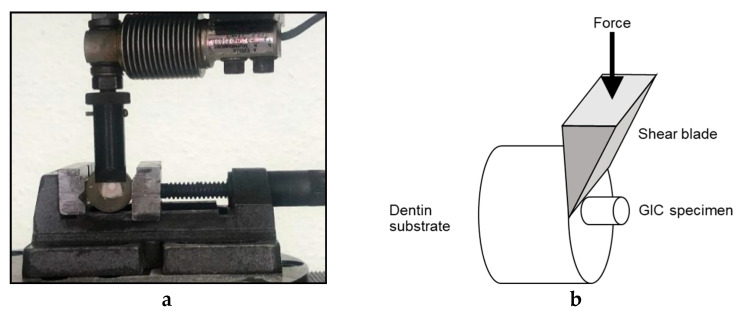
(**a**) Zwick testing machine dislodging GIC specimen; (**b**) Schematic diagram showing shear bond strength testing.

Ninety-seven carious and crack free bovine teeth were selected and stored in NaCl until usage. The teeth were embedded in acrylic blocks. The enamel surface of the teeth was removed using silicon carbide abrasive paper on a polishing machine in order to obtain flat smooth dentin surfaces. Polycarboxylic acid (25%) was used as a dentine conditioner for 25 s then rinsed and air-dried [26,27]. A split teflon mold of a 4 mm diameter and 3 mm height was clamped onto the exposed dentin surface of the tooth using a metallic device with springs and screws for opening and closing (Figure 4). The cement was mixed as per the manufacturer instructions (1:2), packed and condensed inside the mold, and allowed to set. One hour later, the metallic device was opened and the mold was removed, leaving the specimen attached to the dentine. Teeth with the bonded specimens were then stored at 37 °C in deionized water for 24 h. Each specimen was placed in a universal testing machine (Zwick Zmart. Pro, Zwick/Roell, Ulm, Germany) and subjected to dislodging forces at a crosshead speed of 0.5 mm/min using a sharp knife-like mandrel that was attached to the upper assembly (Figure 5a,b). The dislodging force was recorded and then the bond strength of GIC to dentine was calculated according to the following equation [59,60]: shear bond strength [MPa] = force / area.

#### Failure Mode Analysis

**Figure 6:**
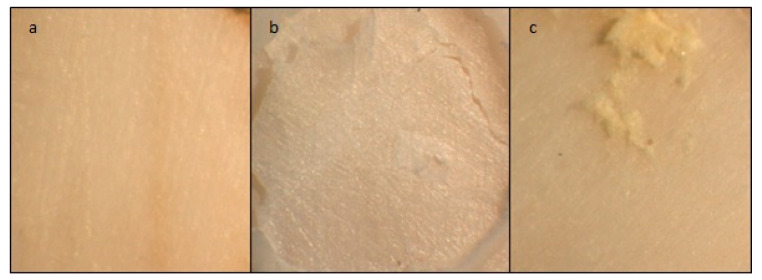
Stereomicroscope images of dentine showing the three failure modes at a magnification of 12×. (**a**) adhesive failure; (**b**) cohesive failure; (**c**) mixed failure.

The different failure modes for all the tested groups (*n* = 10) were evaluated by one observer under an optical microscope (Stereomicroscope SR, Carl Zeiss AG, Oberkochen, Germany) at a 12× magnification. Failure modes were categorized into three groups: adhesive failure when the GIC was removed from the dentin surface without residual debris, cohesive failure when a fracture occurred inside the cement or the tooth, and mixed failure when a combination of both cohesive and adhesive failures was observed (Figure 6a–c) [61].

### 3.3. Film Thickness

**Figure 7:**
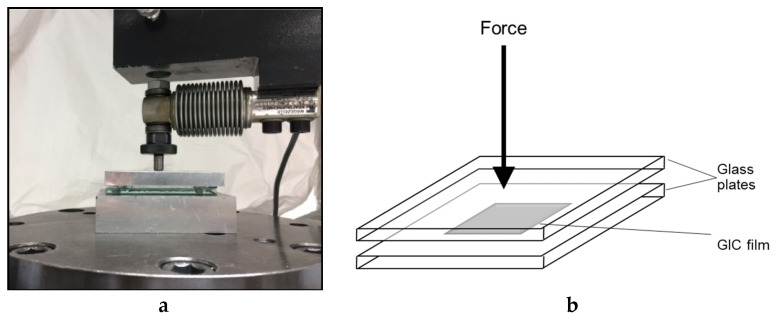
(**a**) Loading of two glass plates with film thickness specimen in between; (**b**) Schematic diagram showing film thickness testing.

The test was conducted according to ISO 9917-1:2007 for glass ionomer cement. The thickness of two flat, uniform, rectangular glass plates stacked in contact was measured four times to the nearest 0.1 µm with a digital micrometer (Digimatic, Mitutoyo Europe GmbH, Neuss, Germany). This reading was recorded as Reading A. The cement for each group (*n* = 10) was prepared according to the manufacturer’s instructions and then a standardized amount of each cement mixture was placed between the two glass plates. A 147 N load was applied on the upper glass plate using a universal testing machine; see Figure 7a,b. Seven minutes later, the overall thickness of the plates with the cement between was recorded as Reading B. The difference between the thickness of the plates with and without the material between (B−A) was considered as the final combined film thickness for the specimen being tested [53].

### 3.4. Statistical Analysis

The Ryan–Joiner normality test (similar to Shapiro–Wilk test) was used to test whether or not the variables followed a normal distribution. The numerical data showed a non-parametric distribution, and thus were presented as a median and interquartile range, *p* ≤ 0.05. Furthermore, the Kruskal–Wallis test was used for comparison between the groups, followed by Dunn’s post hoc test for pairwise comparison. Statistical analysis was performed via Minitab 17.3.1 for Microsoft Windows (Minitab, Inc., State College, PA, USA).

## 4. Conclusions

Within the limitations of the current study, it can be concluded that the addition of a plant extract mixture in an attempt to enhance the antimicrobial activity did not negatively alter the shear bond strength and film thickness properties of GIC, and thus this might have potential for GIC modifications.

## Figures and Tables

**Table 1:** Results of the Kruskal Wallis H test for shear bond strength.

Groups	*n*	Median (MPa)	Interquartile Range	*p* *	Pairwise Comparison **	
Control	20	3.4	3.0	0.046	A	B
CHX	18	1.7	1.4		A	
1:2	21	5.1	7.5			B
1:1	20	3.2	5.6			B
2:1	18	4.3	8.7			B

* Significant at *p* ≤ 0.05. ** Groups that do not share a letter are significantly different.

**Table 2:** Percentages of the different failure modes for each tested group (*n* = 10).

Failure Mode	Control	CHX	1:2	1:1	2:1
Adhesive %	10	15	14	21	0
Cohesive %	50	38	57	36	40
Mixed %	40	46	29	43	60

**Table 3:** Results of the Kruskal Wallis H test for film thickness.

Groups	*n*	Median (μm)	Interquartile Range	*p* *	Pairwise Comparison **	
Control	10	20	2.8	<0.001	A	
CHX	10	20	4.0		A	
1:2	10	22	3.0		A	
1:1	10	22	1.8		A	B
2:1	10	24	1.3			B

* Significant at *p* ≤ 0.05. ** Groups that do not share a letter are significantly different.

## Data Availability

Ethical guidelines were followed strictly.
